# The influence of socioeconomic status on future risk for developing Type 2 diabetes in the Canadian population between 2011 and 2022: differential associations by sex

**DOI:** 10.1186/s12939-015-0245-0

**Published:** 2015-10-24

**Authors:** Laura A. Rivera, Michael Lebenbaum, Laura C. Rosella

**Affiliations:** Public Health Ontario, 480 University Avenue, Toronto, Ontario M5G 1 V2 Canada; Dalla Lana School of Public Health, University of Toronto, 155 College Street, Health Sciences Building 6th Floor, Toronto, Ontario M5T 3 M7 Canada; Institute for Clinical Evaluative Sciences, G1 06, 2075 Bayview Avenue, Veterans Hill Trail, Toronto, Ontario M4N 3 M5 Canada

**Keywords:** Diabetes, Prevention, Socioeconomic status, Risk algorithm, Sex

## Abstract

**Background:**

Articulating future risk of diabetes at the population level can inform prevention strategies. While previous studies have characterized diabetes burden according to socioeconomic status (SES), none have studied future risk.

**Methods:**

We quantified the influence of multiple constructs of SES on future diabetes risk using the Diabetes Population Risk Tool (DPoRT), a validated risk prediction algorithm that generates 10-year rates of new diabetes cases. We applied DPoRT to adults aged 30–64 in the 2011–2012 Canadian Community Health Survey (*n* = 65,372) and calculated risk for 2021–22. A multi-category outcome was created classifying risk as low (≤5 %), moderate (greater than 5 % and less than 20 %), and high (≥20 %), then assessed the impact of individual-level SES indicators, and area-level measures of marginalization on being moderate or high risk using multinomial logistic regression, stratified by sex.

**Results:**

We found nuanced profiles of social determinants by sex, where women are more sensitive to social context. Women living in households where highest educational attainment was less than secondary school were at greater risk [odds ratio (OR) of high compared to low diabetes risk 3.10, 95 % confidence interval (CI) 2.19-4.40, *p* < 0.0001). The same relationship was less pronounced for males (OR 2.17, 95 % CI 1.42-3.32, *p* = 0.0004). Lower household income and being food insecure predicted high future diabetes risk for women (OR 1.37, 95 % CI 1.01-1.86, *p* = 0.0418 comparing quintile 1 to quintile 5; OR 2.64, 95 % CI 1.78-3.92, p < 0.0001 comparing severely food insecure to food secure), but not men (OR 1.15, 95 % CI 0.84-1.57, *p* = 0.3818 and OR 1.22, 95 % CI 0.71-2.10, *p* = 0.4815). At the area-level, material deprivation was significantly associated with increased future risk comparing the most to the least deprived (OR females 2.39, 95 % CI 1.77-3.23; OR males 1.61, 95 % CI 1.22-2.14). Additionally, a strong protective effect was observed for women living in ethnically dense areas (OR 0.75, 95 % CI 0.63-0.89, *p* = 0.0011) which was not as pronounced for men (OR 0.95, 95 % CI 0.76-1.18, *p* = 0.6351).

**Conclusions:**

This study characterized socio-contextual predictors for future diabetes risk, showing sex-specific effects. Diabetes prevention must consider factors beyond individual-level behavioral lifestyle change and actively take steps to mitigate the adverse impacts of socio-contextual factors.

## Background

The rapidly rising burden of type 2 diabetes mellitus represents a burgeoning threat to population health [1, 2] and is associated with significant morbidity, mortality, and economic burden [3]. In Canada, diabetes is the seventh-leading cause of death [4] with 4.2 million cases projected by 2020 [1]. As such, preventative action to curb the tide of diabetes is urgently warranted.

The determinants of diabetes are multifactorial [5, 6]. In understanding disease risk, it is critical to acknowledge broad factors underpinning inequitable distributions of disease such that adverse health is concentrated among the more disadvantaged [7, 8]. Diabetes is more common among the poor and excluded [9, 10], and diabetes risk factors have been shown to be most prevalent among the most socioeconomically disadvantaged [11–17]. Additionally, once diagnosed with diabetes, individuals of lower socioeconomic status (SES) are at greater risk of mortality compared to those of higher SES [10, 18]. Previous work has explored sex-specific patterning of the impact of social position on diabetes risk, with most positing that the association exists more strongly in women than in men [19–22]. Despite these characterizations of the SES-gradients in diabetes risk, explicit consideration of future population-level diabetes risk, a measure that is key in designing effective and impactful diabetes prevention policy, is lacking.

Developing disease prevention strategies includes identifying the level of risk (i.e. the probability of developing diabetes) in the population under study, which can be determined using population-based risk tools. The Diabetes Population Risk Tool (DPoRT) is a validated risk prediction algorithm designed to generate population-level diabetes risk estimates using routinely-collected health survey data [23] and is currently being implemented in public health agencies to inform chronic disease prevention programming [24].

This study aims to elucidate the contextual determinants of high population-level risk for diabetes, taking a diverse perspective on SES by exploring the associations of individual- and area-level constructs of SES with increased diabetes risk. This work takes a novel perspective by characterizing future risk at the population level, making use of a validated population risk algorithm.

## Methods

### Data source

Data was obtained from the Share File of the Canadian Community Health Survey (CCHS) annual component, 2011. The CCHS is a nationally representative cross-sectional survey administered by Statistics Canada that collects information on the health status and determinants of the Canadian population [25]. The CCHS employs a multi-stage stratified cluster design, drawing from a sample of approximately 65,000 respondents representing roughly 98 % of the Canadian population, 12 years of age or older, living in the ten provinces and three territories. The 2 % of individuals not represented by the survey are those who are full time members of the Canadian Forces, and those residing on First Nations Reserves, Crown Lands, in institutions, or in certain remote areas. The CCHS employs three sampling frames to select the sample of households to survey, of which 40.5 % of the sample are selected from an area frame, 58.5 % are selected from a list frame of telephone numbers, and 1 % are selected from a Random Digit Dialling (RDD) sampling frame. Further details regarding the CCHS methodology are documented elsewhere [25].

### The Diabetes Population Risk Tool (DPoRT)

The details on the development and validation of the Diabetes Population Risk Tool (DPoRT) has been developed and validated previously [23, 26]. Briefly, the tool was developed by linking 1996/7 and 2001 respondents to a validated diabetes registry in order to generate survival-based predication equations for diabetes incidence [24]. These equations were subsequently developed across time (using an alternate survey) as well as externally validated in another province. These risk equations have been used to estimate future burden of diabetes in several jurisdictions as well as have been used to assess the impact of diabetes interventions. In this study, we used DPoRT to generate risk probabilities of developing physician-diagnosed diabetes for each respondent of the CCHS 2011. The study population included individuals between 20 and 65 years old, non-pregnant, and not previously diagnosed with diabetes. We restricted the population to individuals 65 years and younger, given that the individual-level and ecological social determinants of health among the working age and non-working-age populations are conceptually different. For example, the elderly are to a greater extent affected by dependency, and taking a life course approach, experience a greater accumulation of socioeconomic disadvantage late in life compared to their younger counterparts. In particular, given the focus of this study on the primary prevention of diabetes, we wished to focus on the non-elderly for whom preventive interventions might have the greatest impact. In this analysis, risk is calculated for a 10-year period, i.e. probabilities generated by DPoRT are for developing physician-diagnosed diabetes within 10 years.

DPoRT was developed using routinely collected, self-reported health behaviour and socio-demographic information from the 1996 National Population Health Survey (NPHS). DPoRT is based on the Weibull survival distribution, a time-to-event model that predicts the probability of developing diabetes in a given follow-up time period by incorporating time in its equation. Sex-specific versions of DPoRT were created to acknowledge that predictors of diabetes are distinct for males and females [27]. Self-reported information required to generate DPoRT estimates include age, sex, body mass index, ethnicity, immigrant status (for women only), education, smoking status, history of hypertension, and history of heart disease. A complete description of the sex-specific DPoRT algorithm formulae and an example of its use for risk calculation is available [23, 26].

### Study variables

The outcome, “DPoRT risk category”, was created using 10-year DPoRT estimates categorized into low (less or equal to 5 % risk), medium (greater than 5 % and less than 20 % risk), and high-risk (greater than or equal to 20 %) groups. The cut-offs employed in creating these risk categories were chosen based on previous empiric studies [26].

We examined DPoRT risk in relation to individual-level and area-level indicators of socioeconomic status (SES). Individual-level explanatory variables included household educational level, equivalized household income quintile, and food security status. The CCHS reports highest household educational attainment in four categories: less than secondary school graduation, secondary school graduation, some post-secondary school, and post-secondary graduation. Food security status as provided by the CCHS has three categories: food secure, moderately food insecure, and severely food insecure. Equivalized household income quintiles were constructed from total household income provided in the CCHS weighted by the number of household members in different age groups. Area-level indicators of SES were obtained using the Canadian Marginalization Index (CAN-Marg) for material deprivation and ethnic concentration [28]. The CAN-Marg material deprivation index measures the relative socioeconomic disadvantage of a census dissemination area (DA) compared to the rest of Canada. The dissemination area is the smallest geographic area at which all census data is disseminated and consists of populations of 400–700 individuals. CAN-Marg is a census- and geographically based index, developed using a theoretical framework based on previous work on deprivation and marginalization and empirically created using principal components factor analysis [28].This index uses six variables from the 2006 Canadian Census: population aged 25 and over without a certificate, diploma, or degree; lone-parent families; population receiving government transfer payments; unemployed population aged 15 and over; population under the low-income cut-off; and households living in dwellings needing major repairs. The CAN-Marg ethnic concentration index was constructed using two Census variables: proportion of recent immigrants, and proportion of visible minorities. CAN-Marg indices were linked to individual-level CCHS Share File data using Statistics Canada’s Postal Code Conversion File Plus. Concepts explored as potential third variables were age, white/non-white ethnicity, immigrant status, self-perceived health, physical activity level, smoking status, and satisfaction with life.

### Statistical analysis

Analyses were carried out separately for women and men. Descriptive statistics were generated to characterize the study population. Multinomial logistic regression was employed to assess the impact of individual- and area-level socioeconomic factors on the categorical DPoRT outcome, providing odds ratios and 95 % confidence intervals. Models were age- and multivariate-adjusted for other demographic and health behaviour- and attitude factors based on public health significance in conceptualizing the relationship between diabetes risk and social context. Fully-adjusted models included continuous age, white/non-white ethnicity, immigrant status, self-perceived health (excellent, very good, good, fair, poor), physical activity level (active, moderately active, inactive), smoking status (three categories, employing Centres for Disease Control criteria: current smoker, former smoker, never smoker) and satisfaction with life (very satisfied/satisfied, neutral, dissatisfied/very dissatisfied).

Results were generated using sample weights provided by Statistics Canada, and bootstrapping techniques were used to calculate variance estimates and confidence intervals. These procedures were employed to account for the sampling methodologies of the CCHS. Analyses were conducted using SAS, version 9.3 (SAS Institute, Cary NC). Ethics approval for this study was obtained through the University of Toronto Research Ethics Board.

## Results

### Study population

Among CCHS 2011 survey respondents, *n* = 65,372 individuals (*n* = 36,030 females, *n* = 29,342 males) were eligible for inclusion and had DPoRT risk estimates generated. The study cohort represents *n* = 9,411,692 females and *n* = 9,405,332 males in the Canadian population. Table 1 provides weighted descriptive frequencies to characterize the study population.

**Table 1:** Characteristics of male and female survey participants, by Diabetes Population Risk Tool risk category (representative of *n* = 9,411,692 females and *n* = 9,405,332 males in Canada)

	Females				Males				Differences between females and males		
	Overall (n, weighted)	Low Risk (%)	Moderate Risk (%)	High Risk (%)	Overall (n, weighted)	Low Risk (%)	Moderate Risk (%)	High Risk (%)	Low Risk	Moderate Risk	High Risk
Socioeconomic status											
Education, Highest attained in household											
Less than secondary	314,654	31.4	49.4	19.2	323,506	27.1	47.2	25.7	*p* = 0.002	*p* = 0.006	*p* = 0.034
Secondary school graduation	854,063	40.6	45.7	13.6	894,214	34.3	45.4	20.3			
Some post-secondary	356,749	49.5	43.0	7.4	341,555	44.3	42.4	13.3			
Post-secondary graduation	7,495,265	58.7	34.8	6.4	7,264,029	42.9	44.0	13.2			
Household income quintile											
Quintile 1 (lowest 20 %)	1,676,952	50.7	39.4	9.9	1,301,012	45.9	39.6	14.5	*p* = 0.030	*p* < 0.001	*p* < 0.001
Quintile 2	1,737,598	52.7	39.0	8.3	1,566,842	40.5	42.6	16.9			
Quintile 3	1,922,377	54.7	37.4	7.8	1,814,805	44.1	41.0	14.8			
Quintile 4	1,986,387	56.4	36.9	6.7	2,199,854	42.6	42.6	14.8			
Quintile 5 (highest 20 %)	2,060,668	62.5	32.0	5.5	2,493,332	38.8	49.3	11.9			
Food security											
Food secure	8,504,214	66.9	29.1	4.0	8,690,076	41.8	44.1	14.1	*p* = 0.066	*p* < 0.001	*p* < 0.001
Moderately insecure	595,581	58.4	35.2	6.4	508,832	46.5	36.7	16.8			
Severely insecure	258,119	47.5	42.1	10.3	163,891	39.2	43.5	17.3			
Demographics											
Ethnicity											
White	7,276,422	59.5	34.3	6.2	7,210,856	43.9	43.8	12.2	*p* = 0.006	*p* < 0.001	*p* = 0.460
Non-white	2,135,270	42.5	45.4	12.1	2,194,476	35.6	43.2	21.2			
Immigrant status											
Yes	7,240,573	59.2	34.5	6.4	7,070,795	43.8	43.5	12.7	*p* = 0.001	*p* = 0.001	*p* = 0.293
No	2,171,119	44.0	44.6	11.4	2,278,513	36.8	44.2	19.0			
Health, behavior, attitudes											
Self-perceived health											
Excellent	2,110,877	68.4	28.9	2.6	2,171,360	53.3	39.6	7.1	*p* < 0.001	*p* < 0.001	*p* < 0.001
Very good	3,918,268	59.9	35.2	4.9	3,917,345	44.2	43.7	12.1			
Good	2,523,097	45.4	42.5	12.1	2,591,761	34.6	46.0	19.4			
Fair	665,897	35.3	47.5	17.2	570,411	24.4	46.2	29.4			
Poor	189,992	34.7	44.3	21.0	141,213	16.7	49.1	34.2			
Total physical activity level (leisure and transportation)^a^											
Active	2,636,812	66.9	29.1	4.0	2,956,217	49.7	44.1	14.1	*p* < 0.001	*p* < 0.001	*p* = 0.002
Moderately active	2,546,977	58.4	35.2	6.4	2,454,829	41.7	36.7	16.8			
Inactive	4,126,345	47.5	42.1	10.3	3,925,487	36.2	43.5	17.3			
Smoking status											
Current	1,942,810	57.3	36.5	6.2	2,444,004	43.5	43.8	12.7	*p* < 0.001	*p* < 0.001	*p* < 0.001
Former	2,163,606	50.0	40.0	9.9	2,381,000	29.1	50.0	20.9			
Never	4,032,660	55.9	36.6	7.5	3,099,374	48.8	39.0	12.2			
Satisfaction with life											
Very satisfied/satisfied	8,676,427	57.2	36.3	6.6	8,717,436	42.7	43.5	13.8	*p* = 0.489	*p* = 0.161	*p* < 0.001
Neutral	414,434	43.9	39.0	17.2	429,938	32.0	47.4	20.5			
Dissatisfied/very dissatisfied	238,404	37.6	42.7	19.7	229,700	35.2	41.8	23.0			
Can-marg indices^b^											
Material deprivation											
Quintile 1 (least deprived	2,283,994	63.4	31.7	4.9	2,257,736	42.4	45.9	11.7	*p* < 0.001	*p* < 0.001	*p* = 0.224
Quintile 2	2,065,506	55.6	36.7	7.6	2,007,398	43.4	42.3	14.3			
Quintile 3	1,910,718	53.7	38.1	8.2	1,892,162	41.2	43.0	15.9			
Quintile 4	1,668,042	52.8	39.0	8.3	1,652,956	42.5	42.1	15.4			
Quintile 5 (most deprived)	1,483,432	49.7	40.6	9.8	1,595,080	40.1	44.8	15.1			
Ethnic concentration											
Quintile 1 (least concentrated)	1,369,771	52.4	39.2	8.4	1,385,597	35.5	47.9	16.6	*p* = 0.026	*p* = 0.230	*p* = 0.028
Quintile 2	1,630,547	56.9	35.5	7.6	1,630,486	39.6	46.0	14.4			
Quintile 3	1,844,042	58.1	36.2	5.7	1,835,057	43.8	41.5	14.6			
Quintile 4	2,105,588	57.3	35.2	7.5	2,035,845	45.7	41.9	12.4			
Quintile 5 (most concentrated)	1,461,744	53.4	38.2	8.4	2,518,346	42.8	42.8	14.4			

^a^Total physical activity level as determined by the Canadian Community Health Survey is derived for respondents based on average daily energy expenditure values (kcal/kg/day) calculated from a series of questions about physical activity (i.e. usual daily activities or occupational-related physical activity), physical activity related to travel (i.e. biking or walking to school or work), and leisure time physical activity (i.e. walking, running, gardening, sports) by the respondent in the past three months [25]

^b^The Canadian Marginalization Index (CAN-Marg) is a census- and geographically based index,developed using a theoretical framework based on previous work on deprivation and marginalization and empirically created using principal components factor analysis [23]

In comparing strata for SES indicators (Table 1), individuals belonging to less-advantaged strata contain higher proportions of individuals in moderate and high-risk diabetes categories, compared to individuals belonging to more-advantaged strata. This is most marked for household educational attainment: for women and men respectively, 19.2 % and 25.7 % of respondents in households with less than secondary school education belonged to the high-risk diabetes category, compared to 6.4 % and 13.2 % of those living in a household where a member graduated from post-secondary school. For both sexes, similar trends occur for household income strata (9.9 % of women and 14.5 % of men in the lowest income quintile had greater than 20 % 10-year risk for diabetes, compared to 5.5 % of women and 11.9 % of men in the highest income quintile) and food security status (10.3 % of severely insecure women were in the high-risk group versus 4.0 % of food secure women; 17.3 % of severely insecure men were at high-risk compared to 14.1 % of food secure men). For area-level CAN-Marg indices, similar trends are observed in comparing the least- and most-materially advantaged strata for proportion belonging to the highest-risk category, where 9.8 % of the most materially deprived women are at high-risk versus 4.9 % of the least deprived. A comparison of the proportion of women in the high-risk group for the most and least ethnically dense quintiles shows no difference (both 8.4 %). In men, the least ethnically concentrated quintile has a higher proportion of individuals at high-risk (16.6 %) compared to 14.4 % of individuals in the most concentrated quintile being at highest risk.

### Associations of individual-level household education, income, and food security with DPoRT risk

Age- and multivariate-adjusted regression results are presented in Tables 2 and 3, detailing associations between individual-level social context indicators and moderate and high-risk categories of DPoRT risk.

**Table 2:** Bivariate^a^ and multivariate-adjusted^b^ odds of moderate (greater than 5 % to less than 20 %) risk of developing diabetes in the next 10 years, according to individual-level education, income, and food security, from a multinomial logistic regression model compared to low-risk (≤5 %)

	Moderate risk			
	Females		Males	
	Age-adjusted	Fully-adjusted	Age-adjusted	Fully-adjusted
Highest level of household education				
Less than secondary	2.37 (1.93-2.92, *p* < 0.0001)*	2.04 (1.63-2.55), *p* < 0.0001*	1.47 (1.10-1.98), *p* = 0.0103*	1.39 (1.01-1.92), *p* = 0.0430*
Secondary graduation	1.87 (1.61-2.17, *p* < 0.0001)*	1.77 (1.50-2.09), *p* < 0.0001*	1.47 (1.20-1.81), *p* = 0.0002*	1.47 (1.15-1.87), *p* = 0.0022*
Some post-secondary	1.82 (1.42-2.33, *p* < 0.0001)*	1.83 (1.42-2.36), *p* < 0.0001*	1.28 (0.95-1.72), *p* = 0.1026	1.34 (0.98-1.84), *p* = 0.0642*
Post-secondary grad	Reference	Reference	Reference	Reference
Household income quintile				
Quintile 1 (lowest)	2.11 (1.84-2.43), *p* < 0.0001*	1.43 (1.22-1.67), *p* < 0.0001*	1.59 (1.30-1.95), *p* < 0.0001*	1.05 (0.84-1.33), *p* = 0.6624
Quintile 2	1.79 (1.57-2.03), *p* < 0.0001*	1.36 (1.16-1.59), *p* = 0.0002	1.67 (1.40-2.00), *p* < 0.0001*	1.25 (1.01-1.56), *p* = 0.0430*
Quintile 3	1.55 (1.37-1.76), *p* < 0.0001*	1.36 (1.19-1.56), *p* < 0.0001*	1.19 (1.02-1.40), *p* = 0.0325*	0.99 (0.82-1.20), *p* = 0.9239
Quintile 4	1.40 (1.23-1.59), *p* < 0.0001*	1.39 (1.20-1.60), *p* < 0.0001*	1.18 (1.01-1.38), *p* = 0.0406*	1.17 (0.98-1.39), *p* = 0.0796
Quintile 5 (highest)	Reference	Reference	Reference	Reference
Food security				
Food secure	Reference	Reference	Reference	Reference
Moderately insecure	1.77 (1.45-2.16), *p* < 0.0001*	1.57 (1.26-1.95), *p* < 0.0001*	1.00 (0.77-1.31), *p* = 0.9767	0.76 (0.56-1.03), *p* = 0.0808
Severely insecure	1.54 (1.22-1.95), *p* = 0.0003*	1.30 (1.00-1.69), *p* = 0.0532	1.40 (0.98-2.00), *p* = 0.0636	1.16 (0.76-1.76), *p* = 0.4885

^a^Bivariate model adjusts for continuous age. ^b^Multivariate model adjusts for continuous age, white/non-white ethnicity, immigrant status, self-perceived health, smoking status, physical activity level, and life satisfaction

*indicates statistical significance at alpha = 0.05

**Table 3:** Bivariate^a^ and multivariate-adjusted^b^ odds of high risk (≥20 %) of developing diabetes in the next 10 years, according to individual-level education, income, and food security, from a multinomial logistic regression model compared to low-risk (≤ %)

	High risk			
	Females		Males	
	Age-adjusted	Fully-adjusted	Age-adjusted	Fully-adjusted
Highest level of household education				
Less than secondary	4.18 (3.10-5.68), *p* < 0.0001*	3.10 (2.19-4.40), *p* < 0.0001*	2.47 (1.72-3.53), *p* < 0.0001*	2.17 (1.42-3.32), *p* = 0.0004*
Secondary graduation	2.80 (2.22-3.53), *p* < 0.0001*	2.84 (2.20-3.67), *p* < 0.0001*	2.18 (1.71-2.76), *p* < 0.0001*	2.22 (1.66-2.98), *p* < 0.0001*
Some post-secondary	1.92 (1.24-2.98), *p* = 0.0037*	2.11 (1.29-3.46), *p* = 0.0029*	1.34 (0.93-1.92), *p* = 0.1182	1.43 (0.94-2.18), *p* = 0.0974
Post-secondary grad	Reference	Reference	Reference	Reference
Household income quintile				
Quintile 1 (lowest)	3.67 (2.84-4.74), *p* < 0.0001*	1.37 (1.01-1.86), *p* = 0.0418*	2.92 (2.24-3.79), *p* < 0.0001*	1.15 (0.84-1.57), *p* = 0.3818
Quintile 2	2.36 (1.81-3.09), *p* < 0.0001*^,¥^	1.38 (1.02-1.87), *p* = 0.0346*	3.28 (2.54-4.23), *p* < 0.0001*^,¥^	1.63 (1.21-2.19), *p* = 0.0013*
Quintile 3	1.97 (1.51-2.57), p < 0.0001*	1.38 (1.04-1.85), *p* = 0.0280*	1.95 (1.54-2.47), *p* < 0.0001*	1.34 (1.02-1.77), *p* = 0.0353*
Quintile 4	1.50 (1.17-1.94), p < 0.0001*	1.36 (1.03-1.79), *p* = 0.0327*	1.86 (1.51-2.30), *p* < 0.0001*	1.57 (1.24-2.00), *p* = 0.0002*
Quintile 5 (highest)	Reference	Reference	Reference	Reference
Food security				
Food secure	Reference	Reference	Reference	Reference
Moderately insecure	2.79 (2.01-3.86), *p* < 0.0001*	1.56 (1.08-2.27), *p* = 0.0194*	1.58 (1.09-2.30), *p* = 0.0161*	1.04 (0.62-1.74), *p* = 0.8839
Severely insecure	4.68 (3.25-6.74), *p* < 0.0001*	2.64 (1.78-3.92), *p* < 0.0001*	1.95 (1.29-2.97), *p* = 0.0017*	1.22 (0.71-2.10), *p* = 0.4815

^a^Bivariate model adjusts for continuous age. ^b^Multivariate model adjusts for continuous age, white/non-white ethnicity, immigrant status, self-perceived health, smoking status, physical activity level, and life satisfaction

*indicates statistical significance at alpha = 0.05; ^¥^indicated female and male estimate significantly differ at alpha = 0.05

In fully-adjusted models, women living in households where the highest educational attainment is less than secondary school graduation had twice the odds of being at moderate risk of developing diabetes in 10 years, compared to women living in households where the highest attainment was post-secondary school graduation [odds ratio (OR) 2.04, 95 % confidence interval (CI) 1.63-2.55; *p* <0.001]. Additionally, women in households with high school graduation has a 77 % increase in odds for being in the moderate risk compared to women in households with post-secondary graduation (OR 1.77, 95 % CI 1.50-2.09; *p* < 0.0001). For men, the associations between household education level and moderate diabetes risk were less pronounced than for women: men in households where highest education was less than secondary graduation were 39 % more likely to be at moderate risk than men in households with post-secondary graduation (OR 1.39, 95 % CI 1.01-1.92; *p* < 0.0430). Models for high-risk diabetes show more marked effects: women in households with less than secondary education having three times the increased odds of a 10-year diabetes risk of 20 % or greater compared to women in households with the most education (OR 3.10, 95 % CI 2.19-4.40; *p* < 0.0001). For men, comparison of the same strata is still large but of smaller magnitude than the relationship in women (OR 2.17, 95 % CI 1.42-3.32; *p* = 0.0004).

Multivariate adjusted models comparing women of lower household income quintiles to the highest quintile demonstrate increased odds of being categorized as moderate risk (Quintile 1 compared to Quintile 5, OR 1.43, 95 % CI 1.22-1.67; *p* < 0.0001) and high risk (Quintile 1 compared to Quintile 5, OR 1.37, 95 % CI 1.01-1.86; *p* = 0.0418). For the female cohort, effect sizes for the odds of being categorized as moderate risk and high risk comparing income strata to the reference group showed less variation than comparisons of educational strata to the highest education category (Tables 2 and 3). Comparing income strata for males, the odds of being at high risk was observed for some comparisons (Quintile 2 compared to Quintile 5, OR 1.63, 95 % CI 1.21-2.19; *p* = 0.0013) but was not significant comparing the most deprived quintile to the reference group (OR 1.15, 95 % CI 0.84-1.57; *p* = 0.3818).

The impact of food security on being at increased risk for diabetes was observed for women but not men in multivariate models. Compared to food secure women, moderately food insecure women were more likely to be in the moderate risk category (OR 1.57, 95 % CI 1.26-1.95; *p* < 0.0001) and the high-risk category (OR 1.56, 95 % CI 1.08-2.27; *p* = 0.0194). Associations were also observed in comparing severely food insecure women to those that are food secure for being categorized as moderate risk (OR 1.30, 95 % CI 1.00-1.69; *p* = 0.0532) and high risk (OR 2.64, 95 % CI 1.78-3.92; *p* < 0.0001).

### Associations of CAN-Marg material deprivation and ethnic concentration indices with DPoRT risk

Tables 4 and 5 provides bivariate age-adjusted and multivariate adjusted multinomial regression result for CAN-Marg indices of interest.

**Table 4:** Bivariate^a^ and multivariate-adjusted^b^ odds of moderate risk of developing diabetes in the next 10 years, predicted by CAN-Marg material deprivation and ethnic concentration indices

	Moderate risk			
	Females		Males	
	Age-adjusted^a^	Fully-adjusted^b^	Age-adjusted^b^	Fully-adjusted^b^
CAN-Marg Material deprivation				
Q1 (least deprived)	Reference	Reference	Reference	Reference
Quintile 2	1.32 (1.15-1.51), *p* < 0.0001*	1.33 (1.15-1.54), *p* = 0.0002	1.07 (0.91-1.25), *p* = 0.4336	1.03 (0.85-1.26), *p* = 0.7371
Quintile 3	1.44 (1.24-1.67), *p* < 0.0001*	1.39 (1.19-1.63), *p* < 0.0001	1.08 (0.93-1.27), *p* = 0.3245	1.07 (0.88-1.28), *p* = 0.5052
Quintile 4	1.60 (1.40-1.82), *p* < 0.0001*	1.51 (1.30-1.76), *p* < 0.0001	1.19 (1.01-1.41), *p* = 0.0376*	1.14 (0.93-1.40), *p* = 0.2019
Q5 (most deprived)	1.78 (1.55-2.04), *p* < 0.0001*	1.64 (1.39-1.94), *p* < 0.0001	1.39 (1.16-1.66), *p* = 0.0003*	1.13 (0.91-1.41), *p* = 0.2537
CAN-Marg Ethnic concentration				
Q1 (least concentrated)	Reference	Reference	Reference	Reference
Quintile 2	0.86 (0.76-0.97), *p* = 0.0111*	0.81 (0.70-0.94), *p* = 0.0041*	0.96 (0.83-1.12), *p* = 0.5972	0.95 (0.80-1.13), *p* = 0.5740
Quintile 3	0.89 (0.78-1.01), *p* = 0.0697	0.84 (0.73-0.96), *p* = 0.0134*	0.83 (0.70-0.99), *p* = 0.0323*	0.86 (0.72-1.03), *p* = 0.1055
Quintile 4	1.01 (0.88-1.15), *p* = 0.9164	0.85 (0.73-0.99), *p* = 0.0326*	0.98 (0.83-1.16), *p* = 0.8281	0.89 (0.74-1.08), *p* = 0.2437
Q5 (most concentrated)	1.23 (1.07-1.41), *p* = 0.0031*	0.75 (0.63-0.89), *p* = 0.0011*^,¥^	1.40 (1.17-1.67), *p* = 0.0002*	0.95 (0.76-1.18), *p* = 0.6351

^a^Bivariate model adjusts for continuous age. ^b^Multivariate model adjusts for continuous age, white/non-white ethnicity, immigrant status, self-perceived health, smoking status, physical activity level, and life satisfaction

*indicates statistical significance at alpha = 0.05; ^¥^indicated female and male estimate significantly differ at alpha = 0.05

**Table 5:** Bivariate^a^ and multivariate-adjusted^b^ odds of high risk of developing diabetes in the next 10 years, predicted by CAN-Marg material deprivation and ethnic concentration indices

	High risk			
	Females		Males	
	Age-adjusted	Fully-adjusted	Age-adjusted	Fully-adjusted
CAN-Marg Material deprivation				
Q1 (least deprived)	Reference	Reference	Reference	Reference
Quintile 2	1.75 (1.35, 2.27), *p* < 0.0001*	1.75 (1.29, 2.37), *p* = 0.0003*	1.47 (1.13, 1.92), *p* = 0.0047*	1.38 (1.02, 1.86), *p* = 0.0348*
Quintile 3	1.99 (1.53, 2.58), *p* < 0.0001*	1.71 (1.29, 2.29), *p* = 0.0002*	1.57 (1.23, 2.01), *p* = 0.0003*	1.58 (1.19, 2.10), *p* = 0.0014*
Quintile 4	2.35 (1.81, 3.06), *p* < 0.0001*	2.03 (1.50, 2.76), *p* < 0.0001*	1.82 (1.43, 2.32), *p* < 0.0001*	1.61 (1.22, 2.14), *p* = 0.0009*
Q5 (most deprived)	2.91 (2.23, 3.78), *p* < 0.0001*	2.39 (1.77, 3.23), *p* < 0.0001*	2.00 (1.54, 2.60), *p* < 0.0001*	1.33 (0.98, 1.81), *p* = 0.0643
CAN-Marg Ethnic concentration				
Q1 (least concentrated)	Reference	Reference	Reference	Reference
Quintile 2	0.88 (0.71, 1.10), *p* = 0.2623	0.80 (0.63, 1.02), *p* = 0.0711	0.90 (0.72, 1.13), *p* = 0.3716	0.93 (0.72, 1.19), *p* = 0.5768
Quintile 3	0.69 (0.55, 0.86), *p* = 0.0011*^,¥^	0.56 (0.43, 0.73), *p* < 0.0001*^,¥^	0.92 (0.73, 1.17), *p* = 0.4912	0.97 (0.74, 1.27), *p* = 0.8240^¥^
Quintile 4	1.21 (0.94, 1.57), *p* = 0.1402	0.93 (0.69, 1.24), *p* = 0.6006	0.95 (0.75, 1.21), *p* = 0.6840	0.79 (0.60, 1.04), *p* = 0.0927
Q5 (most concentrated)	1.63 (1.27, 2.09), *p* = 0.0001*	0.63 (0.47, 0.86), *p* = 0.0040*	1.81 (1.41, 2.32), *p* < 0.0001*	0.66 (0.48, 0.90), *p* = 0.0097*

^a^Bivariate model adjusts for continuous age. ^b^Multivariate model adjusts for continuous age, white/non-white ethnicity, immigrant status, self-perceived health, smoking status, physical activity level, and life satisfaction

*indicates statistical significance at alpha = 0.05; ^¥^indicated female and male estimate significantly differ at alpha = 0.05

Associations were observed for material deprivation with increased likelihood of being categorized as moderate risk and high risk for women, but not men. Effect sizes increase monotonically comparing strata of increased deprivation to the least deprived reference quintile. The largest effect size occurs in comparing the most deprived quintile to the least deprived, indicating 64 % increased likelihood of being at moderate risk (OR 1.64, 95 % CI 1.39-1.94; *p* < 0.0001) and over two times increased risk of being at high risk (OR 2.39, 95 % CI 1.77-3.23; *p* < 0.0001).

A protective effect was shown for the most ethnically concentrated areas compared to the reference category on being at moderate risk (OR) and high risk (OR). The strongest protective effect was seen comparing the most ethnically concentrated quintile of women to the least concentrated for being at moderate risk (OR 0.75, 95 % CI 0.63-0.89; *p* = 0.0011) and high risk (OR 0.63, 95 % CI 0.47-0.86) for developing diabetes in the next 10 years. For males, a protective effect was seen only in comparing the most concentrated quintile to the reference group for being at high risk (OR 0.66, 95 % CI 0.48-0.90; *p* = 0.0040).

## Discussion

This study explored the role of individual-level and area-level social context indicators with future diabetes risk. We found nuanced profiles of social determinants for women and men, where women are more sensitive to social context including lower household-level education, household income, food insecurity, and living in materially deprived areas, whereas men are affected by lower household education only. Additionally, a strong protective effect was observed for women living in ethnically dense areas, which was not as pronounced for men.

### Mechanisms

Previous Canadian research has found lower educational achievement level to be associated with increased prevalence of Type 2 diabetes [29]. We found diabetes increased monotonically with lower education levels in females, whereas for males only those in the lowest educational strata were more likely to develop diabetes. Education level is a useful proxy for SES, where those with increased attainment are generally employed in professions with higher remuneration, which in turn provides the means to live healthily including resources for healthy food, housing in a safe neighbourhood, opportunities for physical activity, and the lack of a precarious financial situation reduces stress and allostatic load. In attaining formal education, individuals are taught methods for obtaining and appraising information which can benefit their ability to integrate concepts central to healthy living. The differential effects by sex may also be attributed to the decreased return on educational investment for females that has persisted over decades [30].

We found lower household income, food insecurity, and area-level material deprivation to be important determinants of high risk in women, but not men, consistent with income gradients seen in other Canadian analyses [31]. As an indicator of SES, income directly reflects resources available and thus a lack of resources can impede optimal health. For example, a situation of chronic precarious employment and financial instability shifts one’s priorities away from being able to plan and prepare nutritious meals and engage in physical activity [32, 33]. The association detected between CAN-Marg material deprivation, an indicator that encompasses various facets of disadvantage, and higher risk corroborates the aforementioned phenomena from an ecological standpoint, supported in previous work that looked at other area-level characteristics (crime, population and retail density) and found a positive association with diabetes prevalence [34]. Furthermore, low-income females have been found to be less likely to engage in active leisure activities [35]. In the case of food insecure women, household food shortages have been documented as a major barrier to implementing nutrition guidelines; when tasked with the responsibility of providing meals for dependents, women will sacrifice optimal nutrition of their own meals to those in the household [35].

Our results indicate that living in ethnically concentrated areas is a protective factor for diabetes high risk, with larger effect sizes in females. These results provide evidence of the “ethnic density effect”, whereby health benefits are conferred for individuals of ethnic minorities living in areas with dense ethnic populations [36]. Although materially deprived areas are often those that are ethnically concentrated, the protective effects observed in our analyses speak to the potential positive impacts of social cohesion, increased self-evaluation, and mitigated effects of racism [36]. Previous American work has shown the protective association between ethnic density and mental health, noting the role of social support [37, 38]. Isolating the contributions of social capital, neighbourhood trust, reciprocity, and networks in mitigating adverse health remain to be investigated further [36, 39].

### Study limitations

A potential limitation of the outputs generated by DPoRT is the tool’s reliance on self-reported health information as algorithm inputs to generate risk probabilities. Self-reported health information is subject to misclassification and can be influenced by respondent characteristics such as knowledge of the health issue, recall ability, and social desirability bias [40]. Furthermore, these tendencies are likely patterned by sex. In the case of DPoRT, there is evidence that clinical risk tools that incorporate self-reported data perform as well as models that do not [41, 42]. Indeed, the tool’s basis on self-reported risk factors, in fact, increases the accessibility of the tool while maintaining accuracy.

### Implications

This study contributes to the evidence informing our understanding of how socioeconomic status at the individual and ecological levels shape increased risk for diabetes. These findings should be considered by the diabetes prevention and management community, and by decision-makers who might influence the political and economic structures that give rise to the socio-contextual conditions that contribute to increased risk for chronic diseases such as diabetes. In this age of rapidly rising chronic disease burden, primary prevention strategies must go beyond the status quo promotion of healthy lifestyles that target individuals. In light of these findings, prevention strategies must include actions to mitigate the adverse impacts of socio-contextual factors that contribute to diabetes risk, which includes the consideration of health in all policies, the modification of social and economic structures that create inequality, and the empowerment of those in positions of disadvantage [43]. Under the status quo, diabetes has risen dramatically; a paradigm shift in the diabetes prevention and management community is warranted.

## Key messages

- Articulating population-level future diabetes risk informs primary prevention strategies.
- Women are more sensitive to social context with respect to future diabetes risk.
- Area-level material deprivation predicts high risk, while ethnic concentration is protective.
